# Hox Gene Expression Leads to Differential Hind Leg Development between Honeybee Castes

**DOI:** 10.1371/journal.pone.0040111

**Published:** 2012-07-25

**Authors:** Ana Durvalina Bomtorin, Angel Roberto Barchuk, Livia Maria Moda, Zila Luz Paulino Simoes

**Affiliations:** 1 Departamento de Genética, Faculdade de Medicina de Ribeirão Preto, Universidade de São Paulo, Ribeirão Preto, São Paulo, Brazil; 2 Departamento de Biologia Celular, Tecidual e do Desenvolvimento, Instituto de Ciências Biomédicas, Universidade Federal de Alfenas, Alfenas, Minas Gerais, Brazil; 3 Departamento de Biologia, Faculdade de Filosofia, Ciências e Letras de Ribeirão Preto, Universidade de São Paulo, Ribeirão Preto, São Paulo, Brazil; Arizona State University, United States of America

## Abstract

Beyond the physiological and behavioural, differences in appendage morphology between the workers and queens of *Apis mellifera* are pre-eminent. The hind legs of workers, which are highly specialized pollinators, deserve special attention. The hind tibia of worker has an expanded bristle-free region used for carrying pollen and propolis, the corbicula. In queens this structure is absent. Although the morphological differences are well characterized, the genetic inputs driving the development of this alternative morphology remain unknown. Leg phenotype determination takes place between the fourth and fifth larval instar and herein we show that the morphogenesis is completed at brown-eyed pupa. Using results from the hybridization of whole genome-based oligonucleotide arrays with RNA samples from hind leg imaginal discs of pre-pupal honeybees of both castes we present a list of 200 differentially expressed genes. Notably, there are castes preferentially expressed cuticular protein genes and members of the P450 family. We also provide results of qPCR analyses determining the developmental transcription profiles of eight selected genes, including *abdominal-A*, *distal-less* and *ultrabithorax* (*Ubx*), whose roles in leg development have been previously demonstrated in other insect models. *Ubx* expression in workers hind leg is approximately 25 times higher than in queens. Finally, immunohistochemistry assays show that Ubx localization during hind leg development resembles the bristles localization in the tibia/basitarsus of the adult legs in both castes. Our data strongly indicate that the development of the hind legs diphenism characteristic of this corbiculate species is driven by a set of caste-preferentially expressed genes, such as those encoding cuticular protein genes, P450 and Hox proteins, in response to the naturally different diets offered to honeybees during the larval period.

## Introduction

*Apis mellifera* queens and workers are prime examples of how deeply the environment can affect ontogenesis. These two classes of females, named castes, develop from genetically equivalent eggs that undergo different developmental pathways in response to different diets, thus constituting an example of the widespread phenomenon of developmental plasticity. Processes and concepts associated with this phenomenon have attracted researchers’ attention over time. Despite this interest, the genetic cascade linking nutrition to the morphological outputs in such divergent and specialized organisms is unknown and represents a very interesting biological problem.

The development of complex traits, such as wings and other appendages, is strongly influenced by nutrition and population conditions [1]. In bees, a differential protein-containing diet is responsible for the high levels of juvenile hormone (JH) observed in queens, which, in turn, directs larval development and the morphological differences observed in both castes [2]. JH has been described as one of the major components of insect development, integrating reproduction and the development of morphological traits. However, in the honeybee *A. mellifera*, JH seems to have lost its gonadotropic activity (for review see [3]), although JH is still capable of activating the expression of specific developmental gene pathways that end up in two specialized morphs, queens and workers [4]. The specific morphological traits and related physiology developed during the differentiation process convert the queen to an organism specialized in reproduction by losing some organ functions and gaining others, whereas workers develop into multitasking and facultatively sterile organisms [5]. A good example of developmental plasticity in honeybee castes is the differentiation of the hind tibia. In workers, this region forms the corbicula, or pollen basket, a smooth region surrounded by a row of long scopal hairs used for carrying pollen and other materials to the nest [6]. The corbicula and corresponding behavior are absent in queens.

Grafting experiments at different times of larval development and the suppression of *tor* (*target of rapamycin*) activity showed that the development of the pollen-collecting apparatus is determined after the fourth larval stage [7], [8] and is probably under the control of JH mutti [9]. Nonetheless, the morphological aspects of the differential development of hind legs in honeybee castes and the molecular mechanism underlying the morphogenesis of the corbicula, which is a morphological characteristic with evolutionary implications because it is synapomorphic for a fundamental branch in bee phylogeny (i.e. the corbiculate Apidae), are still unknown [10].

Here, we show details of the initial steps of hind leg morphogenesis in honeybee castes. We present a list of differentially expressed genes generated by an analysis of the whole genome using hybridization of oligonucleotide arrays with RNA samples from hind leg imaginal discs of pre-pupa of both castes. We used qPCR analyses to validate eight selected genes, including *abdominal-A* (*abd-A*), *distal-less* (*dll*) and *ultrabithorax* (*Ubx*), for which roles in leg development in other insect models have been previously demonstrated [11]–[13], and *ataxin-2* (*atx-2*), *cryptocephal* (*crc*), *dachshund* (*dac*) and *grunge* (*gug*), which are also related to leg development and have been found to be differentially expressed in whole larval samples of both castes [4]. Finally, using immunohistochemistry, we show that the expression of *Ubx* in developing honeybee hind legs is negatively correlated with bristle distribution in the corbicula. These results indicate that a differential nutrition during the initial stages of post-embryonic development leads to the establishment of differential gene expression patterns, including the caste-specific transcription and translation of a Hox gene which seems to be a key player during the differential development of hind legs in honeybee castes.

## Results

### Morphological Analyses

**Figure 1:**
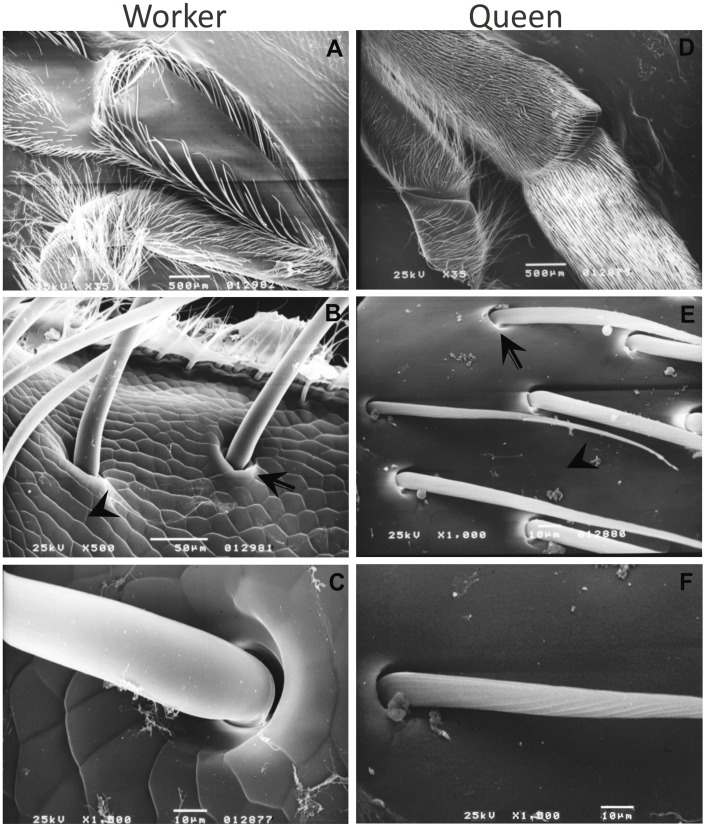
SEM images showing divergent morphologies of *A. mellifera* worker and queen hind legs during pupal development (Pb). **A:** Worker hind leg external surface, note the bristle arrangement forming the pollen basket in the tibia, i.e., corbicula. **B:** Distal portion of the tibia of worker hind leg external surface. **C:** The single bristle on the worker hind leg external surface. This bristle may be a mechanoreceptor like the other bristles on the tibia of the worker hind legs. **D:** Queen hind leg external surface. **E:** Distal portion of the tibia of the queen hind leg external surface. **F:** A bristle on the queen hind leg external surface. Arrow points to bristle socket and arrowhead points to the structure of the cuticle. Original scale bars of scanning electron microscopy system.

The differences in hind leg morphology between castes of adult honeybees, meaning bristle patterning [14] and the stage in which the developmental determination of these caste-specific structures takes place are widely known [7], [8]. To determine the stage the bristles are formed we dissected hind legs from worker and queen pupae for Scanning Electron Microscopy (SEM) analyses. We found that all bristles are formed and correctly positioned in worker and queen hind legs in brown-eyed pupae (Pb) just after apolysis (Fig. 1A and Fig. 1D). Interestingly, we found that the cuticle of worker hind legs is formed by polygonal scales, which contrasts with the smooth appearance of the same region in queens (compare Fig. 1B and 1E, arrowhead). In addition, bristles of the tibia in worker hind legs have a characteristic socket, usually observed in mechanoreceptors (external proprioceptors). These bristles differ strikingly with the bristles found on queen legs, which do not contain this socket aspect [15] (Fig. 1B and 1E, arrow; Fig. 1C and 1F, detail).

### Microarray Analyses

**Figure 2:**
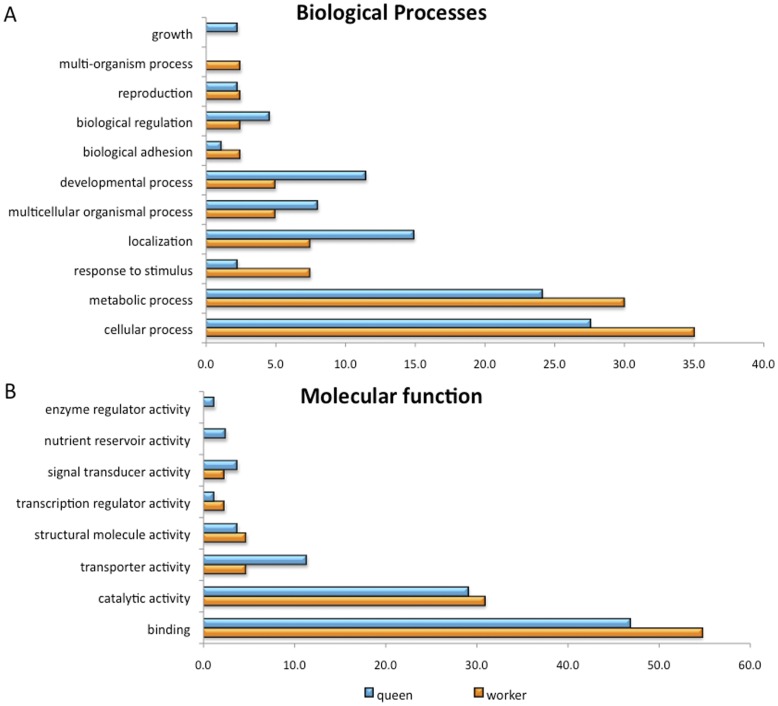
Overrepresented Gene Ontology terms for the differentially expressed genes in the hind legs of worker and queen pre-pupae. **A:** Classification according to Biological Processes at GO. **B:** Classification according to Molecular Function. Blue: queens; Orange: workers.

We performed oligonucleotide microarray hybridization analyses comparing RNA samples from hind leg imaginal discs of queen and worker pre-pupae, which is the stage when the JH level in queens are much higher than that in workers [16]. We got a list of 200 differentially expressed genes (DEGs; see M&M Section, and Table S1). The majority of them were found to be up-regulated in queen pre-pupae (127), whereas 72 were up-regulated in worker pre-pupae (see Table S1 and GEO database, accession number GSE34293). Sixty-five queens’ and 39 workers’ DEGs have orthologs in *Drosophila melanogaster*. Using the Gene Ontology tool of the *D. melanogaster* database, we showed that most of the DEGs that have *D. melanogaster* orthologs code for binding proteins (60%Q/47%W). The second-most represented molecular function in queens belonged to the class of nucleotide binding, ion binding and proteins with hydrolase activity (21%), and in workers, the orthologs were genes coding for proteins with oxidoreductase activity (22%) (Fig. 2).

Among the DEGs detected in queen pre-pupae, we identified the *Drosophila* ortholog *Usp7*, GB17081, which is expressed 18 times more in this caste than in workers. In addition, in this caste we found groups of highly expressed genes clearly associated with JH metabolism, such as *Retinoic and fatty acid Binding protein* (*RfaBp*) and *juvenile hormone acid methyl transferase* (see Table S1). Three other genes, members of the IIS/TOR pathway [*insulin-like peptide-3* (*ILP3*), *tuberous sclerosis protein 1* (*TSC1*) and *Phosphatidylinositol 3-kinase* (*Pi3k*)], as well as three storage proteins, *hexamerins 70a*, *70b* and *110*, were also found to be highly expressed in queen pre-pupa hind legs.

Five members of another group of genes, the Cytochrome P450 (*Cyp*) family, were found to be differentially expressed between castes. Three of them were expressed more in workers (GB18872; GB14915 and GB11973) and two in queens (GB15634 and GB17588). In addition, a homeobox gene related to sensory organ development, *rough* (*ro*), which controls photoreceptor differentiation [17], is over-expressed in workers. Interestingly, *fushi tarazu transcription factor-1* (*ftz-f1*) is expressed at a higher level in queen hind legs. We found three genes up-regulated in workers that are inhibited by Ftz-f1 in honeybees (GB13457, Glycine-rich protein; GB11973, another member of the Cytochrome P450 family related to nervous system development; and GB15203, a cuticular protein gene). A different cuticular protein gene, GB15046, expressed more in queens, is up-regulated by Ftz-f1 (Simoes, ZLP, unpublished data) (Table S1). Overall, we found six cuticular protein genes differentially expressed between castes, three up-regulated in queens (GB30200; GB30334 and GB15046) and three in workers (GB12524; GB15203 and GB13457).

### The Developmental Transcription Profile of the *abd-A*, *atx-2, crc, dac, dll, gug, RfaBp* and *Ubx* genes During Leg Morphogenesis in Honeybee Castes

Because Dedej and colaborators [8] and Patel and colaborators [7] demonstrated that hind leg determination in honeybees occurs between the 4^th^ and 5^th^ larval stages and our results of morphological analyses showed that hind leg structures are completely formed in Pb, we determined the developmental transcription profiles of eight genes associated with leg morphogenesis in honeybee castes from L4 to Pw (Table 1). We chose four genes related to leg development that are up-regulated in whole bodies of fourth larval instar workers (*atx-2*, *crc*, *dac* and *gug;* [4]: *RfaBp,* a gene found to be up-regulated in queen pre-pupa hind legs in this work (Table S1) and previously characterized as down-regulated by JH [4], and the homeobox genes *abd-A*, *dll* and *Ubx,* whose participation in hind leg and bristle leg formation has previously been demonstrated in *D. melanogaster* and hemimetabola [11], [18].

**Table 1:** Characteristics of stages of female honeybee larval development.

Stage of development	Symbol	Characteristic	
Fourth instar larvae	L4	0.004 to 0.0248g (W)	0.004 to 0.044g (Q)
Fifth instar larvae feeding	L5F	0.06 to 0.11g (W)	0.09 to 0.18g (Q)
Fifth instar larvae defecting	L5S	Larval period after sealing	
Prepupae	L5PP	No feeding, inactive stage	
White-eyed pupae	Pw	Pupae with no pigmentation	
Brown-eyed pupae	Pb	Pupae with brown eyes and no pigmentation in the thorax	

W: workers;

Q: queens.

**Figure 3:**
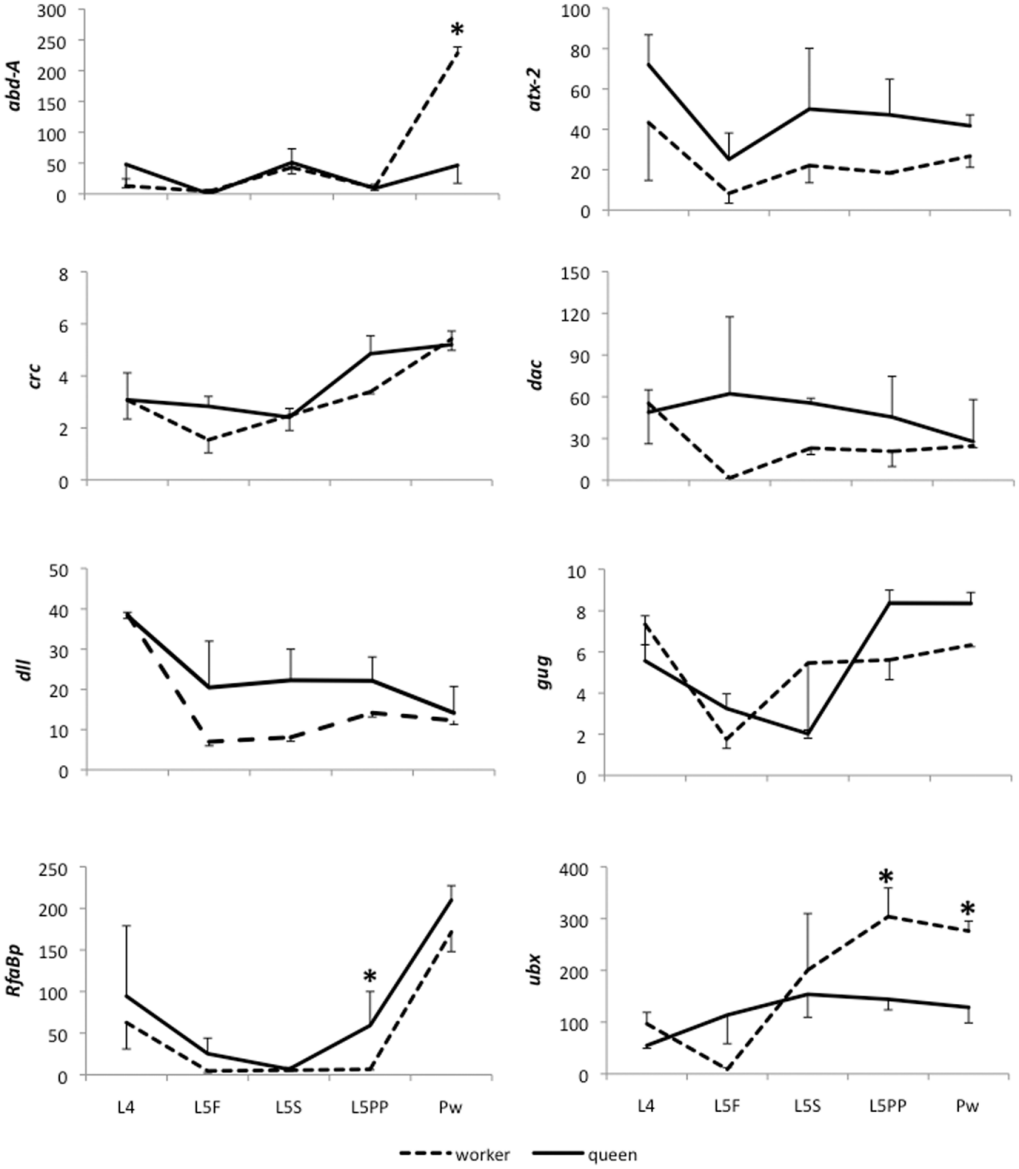
Transcriptional pattern of *abdominal-A*, *ataxin-2*, *cryptocephal*, *dachshund*, *distal-less*, *grunge*, *Retinoic and fat acid Binding protein* and *ultrabithorax* during leg development in *A. mellifera* castes. Ordinates represent relative transcript levels assessed by qRT-PCR. Data were normalized by *ribosomal protein-49*. Three biological samples were analyzed in technical duplicates. L4, L5F and L5S: larval stages; L5PP: pre-pupae; Pw: white-eyed pupae; *: significant statistical differences between castes (P<0.01).

We assessed the transcription profiles of the *abdominal-A*, *ataxin-2, cryptocephal, dachshund, distal-less, grunge* and *ultrabithorax* genes in L4, L5F, L5S, L5PP and Pw (see Table 1) of worker and queen hind leg imaginal discs/legs using Real Time RT-PCR (Fig. 3). Despite its low level of transcription, *abd-A* is similarly expressed in both castes when developmental timing is considered, although it is expressed five times more in the hind legs of worker Pw compared to those of queens (P<0.01). *atx-2* and *dll* also show similar profiles in both castes, with queens expressing higher mRNA levels. However, this difference is not statistically significant (P>0.01). In contrast, *crc* and *gug* do not have similar transcription profiles and are not differentially expressed between castes (P>0.01). *dac* shows a high level of transcripts in queen larval imaginal discs, but this difference is not statistically significant (P>0.01). *RfaBp* shows the same transcription profile in both castes, with levels being slightly higher in queen pre-pupa hind legs (P<0.01). Although *atx-2*, *crc*, *dac* and *gug* are preferentially expressed in the whole body of worker larvae [4], they are not differentially expressed during hind leg development. These genes might play different developmental roles in tissues and organs during development [19]–[23], however during leg development they may be part of the basic leg patterns in both castes and, therefore, are independent of the environment.

Unlike the other genes evaluated, *Ubx* was clearly expressed differently between castes, with higher expression in workers during pre-pupa and white-eyed pupal stages than in queens during the same stages (P<0.01). These results suggest that *Ubx* could regulate differences in hind leg morphological development between queens and workers.

### Immunolocalization of Ubx in the Developing Hind Legs of Queen and Worker Honeybees

**Figure 4:**
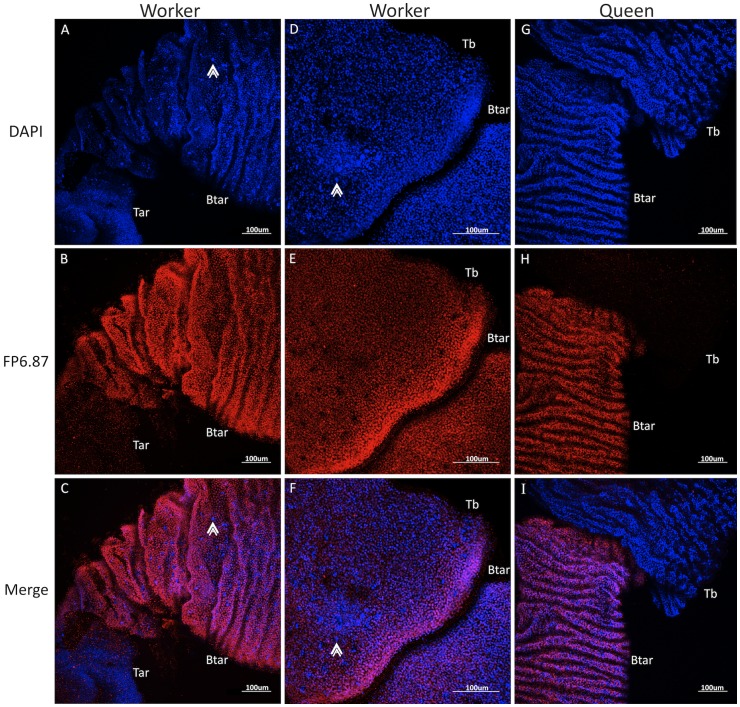
Immunolocalization of Ubx (FP6.87 antibody) in honeybee prepupal hind legs. **A-F:** Ubx is expressed in the tibia and basitarsi of worker pre-pupae. Note that nuclei that do not express Ubx are arranged in a similar pattern to that of bristles in the adult hind leg (arrowhead) (see Figure 1D). **G-I:** Ubx is expressed only in the basitarsi of queen pre-pupae hind legs. In blue: DAPI; in red: Ubx; Tar: tarsi; Btar: basitarsi; Tb: tibia. Original scale bars of confocal system.

In different *Drosophila* species, Ubx regulates, on a fine scale, the differences in bristle and trichome distribution and the morphology of these structures in the hind and middle legs [13], [24]. To compare the distribution of Ubx expression in the hind legs of queens and workers, we performed immunocytochemistry staining using a antibody that detects conserved epitopes in Ubx and Abd-A proteins, the mouse monoclonal antibody FP6.87 [25], and DAPI. At the pre-pupal stage, our results showed that the Ubx protein is localized in two segments of the worker hind leg, whereas in queens, it is localized in only one. Specifically, in workers Ubx is localized to the nucleus of cells in the tibia and basitarsus (Fig. 4B and Fig. 4E), whereas in queens, Ubx is localized only in the basitarsus (Fig. 4G-I).

In the tibia and basitarsus of workers, during pre-pupal stage, some cells are arranged in pairs and the cells are negative for Ubx; these nuclei are larger than the other nuclei in the appendage (Fig. 4A-F). This arrangement is similar to the nuclei of bristle precursor cells, as described by Thurm [26]. However, in queen pre-pupa hind legs, there is no paired arrangement of cells.

**Figure 5:**
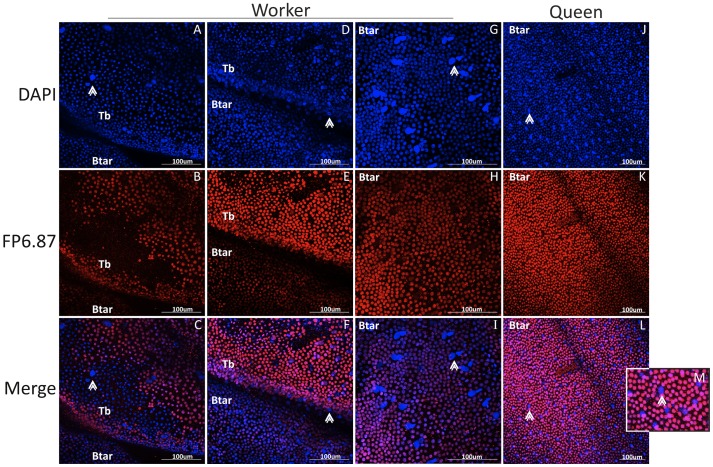
Immunolocalization of Ubx (FP6.87 antibody) in honeybee white-eyed pupale hind legs. **A-C:** Ubx is expressed in the tibia and basitarsi of workers. There is a region in the tibia (which may be the future corbicula) that does not express Ubx. **D-I:** In the basitarsus and distal portion of the tibia (arrowhead) in workers, there are double nuclei that do not express Ubx, arranged in a similar pattern as that of the bristles in the adult hind leg. **J-L:** In the hind legs of queen white-eyed pupae, Ubx is expressed only in the basitarsi. In blue: DAPI; in red: Ubx; Btar: basitarsi; Tb: tibia. Original scale bars of confocal system.

**Figure 6:**
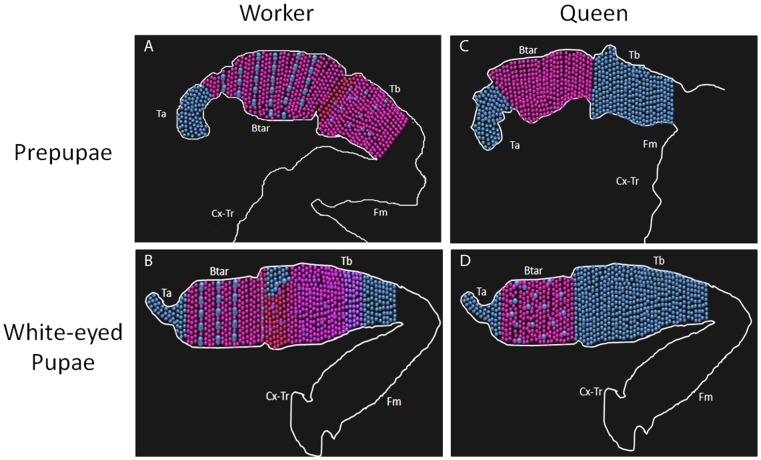
Chart of immunolocalization of Ubx in hind legs of pre-pupae and white-eyed pupae of honeybee castes. **A:** pre-pupae workers’ hind leg; **B:** white-eyed pupae workers’ hind leg; **C:** pre-pupae queens’ hind leg; **D:** white-eyed pupae queens’ hind leg. In blue: DAPI; in red: Ubx, note that the different red degrees represent the Ubx level observed in the stainings; Tar: tarsi; Btar: basitarsi; Tb: tibia; Fm: femur; Cx-Tr: coxa and trochanter.

In early pupae (Pw) worker hind legs, Ubx is still localized in both the tibia and basitarsus, but it is more evident in the tibia. In the tibia, the previously mentioned paired arrangements of the nuclei express Ubx (Fig. 5A-F). However, in the border of the tibia/basitarsus there is a region negative for Ubx. On the other hand, in the basitarsus, the coupled cells present larger nuclei when compared to the other cells present in same leg segment and do not express Ubx (Fig. 5G-I). In Pw queens, Ubx is expressed only in the basitarsus. Opposite to the previous stage (pre-pupal stage), at pupal stages it is easy to see the paired cell arrangement with one of the nuclei negative for Ubx (detail, Fig. 5M). The distribution of the paired cells in queens and workers resembles the adult hind leg bristle distribution. The pattern of expression of both castes, at pre-pupal and pupal stages, is represented in the cartoon Fig. 6.

## Discussion

### Bristles on the Tibia of Worker Hind Legs May be Mechanoreceptors

We show here that the bristles of the tibia in the worker hind legs have characteristics suggesting that they are mechanoreceptors. The morphological organization of this type of bristle has been characterized in honeybees by Thurm, [27] working with head mechanoreceptors. A mechanoreceptor (a type of sensillum) consists of cuticular components, a sensory neuron and a sheath of cells, and it is used for the mechanical perception of external stimuli [28]. Any mechanical force exerted on this type of sensory bristle activates nerve endings [27].

Our microarray analysis evidenced three genes related to the development of the sensorial system as up-regulated in the legs of worker prepupae. Two are members of the P450 family (Cyp303a1 and Cyp4g15). One of these is described as related to the development and function of sensory organs in *Drosophila* [29], and the second as associated with nervous system development [30]. The third gene is annotated as a homeobox gene related to sensory organ development, *ro*, which controls photoreceptor differentiation [17]. The inference is that genes involved in sensory system development are up-regulated in the development of worker hindlegs. This is supported by our SEM results which show that bristles localized to the tibia of worker hind legs have characteristics of sensory organs, whereas the bristles on queen hind legs are much simpler structures. Information on the pollen load on the corbicula would then be conveyed by these mechanoreceptors informing a pollen forager when it is time to return to the hive. Though while plausible, this is still a hypothesis that needs further testing.

### Two Sets of “Caste-specific” Genes may Underlie Differential Cuticular Morphogenesis and Allow for Pollen-collecting Behavior in Workers

Our SEM results also showed that the cuticle that covers the adult hind leg has caste-specific characteristics. In workers, polygonal scales form the cuticle in this region, whereas in queens, it has a smooth appearance (see Fig. 1). This cuticle diphenism might be controlled by the differential expression of cuticular protein genes because queens and workers up-regulate different sets of these genes. This differential expression of cuticular protein genes may be governed by JH acting through the transcription factor Ftz-f1, which, as we could show, is more expressed in queens than in workers. Ftz-f1, an orphan nuclear receptor, is known to activate cuticular protein genes in *Drosophila* [31], [32]. In *A. mellifera*, JH induces the expression of *ftz-f1* in queen-destined larvae, thus possibly driving the expression of cuticular protein genes also in this species. In fact, *ftz-f1* controls the expression of honeybee cuticular protein genes, up-regulating the expression of the GB15046 gene and down-regulating GB15203 (Simoes, ZLP, unpublished data). During pre-pupal development, *ftz-f1* is more expressed in the legs of queens, during a time window when JH titers are up to three times higher in queens than in workers [16]. We could furthermore show that *ftz-f1* and the gene GB15046 are more expressed in queens and that GB15023 is highly expressed in workers at this same time of development. We consider that such differences could explain the differential cuticle formation in the two castes.

As said, we expect the cuticular proteins, which are differentially expressed between castes to be associated with the different cuticle types. Among the cuticular proteins, the CPR family, characterized by the R&R consensus motif [33], is the most abundant one [34]. This family is subdivided into three classes according to the motif present in the consensus region (RR-1, RR-2, RR-3). Whereas RR-1 proteins are related to soft/flexible cuticles and RR-2 proteins to hard/stiff cuticle, the RR-3 proteins cannot be associated with any particular type of cuticle [35]. Using the Cuticle DB [36], classification of the cuticular protein genes expressed during leg development in honeybee castes shows that workers up-regulate two RR-2 genes, whereas queens up-regulate only one RR-1 and another CPR gene that could not be further classified through its *Drosophila* ortholog [37]. Thus, the over-expression of RR-2 genes in workers and the RR-1 gene in queens might be responsible for the rough cuticle of the former and the smooth cuticle of the latter. The rough surface of the worker hind leg tibia (cuticle with polygonal scales), together with the pollen basket (see Fig. 1), pollen comb and pollen press, could be biological stratagems allowing efficient pollen-collecting behavior. It is worthy of note that the corbicula is an ancestral character, present already in solitary species of the corbiculate clade, and conserved in the worker caste of these eusocial bees.

### *Ultrabithorax* Expression Pattern During Hind Leg Development Coincides with Bristle Localization in Adults

Ubx belongs to a family of transcriptional regulators that trigger differential developmental programs along the antero-posterior axis of bilaterian animals [38]–[40]. Besides conservation in sequence and expression domains, changes in Hox gene expression is known to give rise to new structures or even new body patterns during animal evolution, directly linking Hox gene activity with morphological diversity [41]–[44]. *Ubx* expression is known to be determined by other transcription factors and auto-regulation [45]–[46], as well as by epigenetic factors of chromatin modification, with Polycomb and Trithorax being amongst the best known *Ubx* regulators [47]. In *Drosophila,* the ubiquitin-specific protease 7 (*Usp7*) has also been associated with *Ubx* expression, with Usp7 being described as a Hox gene transcription inhibitor that, when disrupted, produces strong homeotic transformations in the second thoracic segment resembling Ubx over-expression [48]. Herein we showed that *Usp7* is preferentially expressed in queen prepupae (microarray experiment). These have lower *Ubx* mRNA and protein levels, pointing towards *Usp7* as a candidate regulator of *Ubx* expression during postembryonic development of honeybees.

We also showed that *Ubx* transcripts and its protein product are differentially expressed in queens and workers of *A. mellifera* during the development of caste- specific hind leg morphologies. A detailed analysis of specific segments of worker hind leg morphology revealed that the pollen basket is formed in a region of the tibia that is free of bristles or trichomes, and it is there where we detected high levels of Ubx in prepupal and early pupal phases. The basitarsus of adult workers shows a linear arrangement of bristles, the pollen comb, and is exactly in this region were Ubx expression was absent in certain cells with large nuclei during early pupal stages of workers. Furthermore, the hind legs of queens are covered with bristles and Ubx expression was absent in the respective tibia segment. The distribution of bristles and trichomes and their presence/absence on the hind leg are also controlled by Ubx expression in *Drosophila melanogaster* [24] and in related species (*D. simulans* and *D. virilis*), where an interesting polymorphism was observed on the femur of the hind leg, “the naked valley”[24]. This region is characterized by high levels of Ubx expression, which is not observed in other species of the group where this region of the leg is covered by trichomes, leading to infer that development of the “naked valley” is dependent on the absence of Ubx expression [24]. This region may thus be considered as ontogenetically equivalent to the pollen basket on the tibia of *A. mellifera* workers.

The point of bristle insertion in the cuticula, the socket, also revealed an important trait. In the worker tibia it has a mechanosensorial-type morphology, similar to that described in *Drosophila,* that was correlated with Ubx localization in different bristle precursor cells in late larval and early pupal stages [13]. In worker honeybees, the localization of Ubx also appears to be associated with the mechanosensorial character of these bristles. These data strongly suggest that the differential expression of Ubx is associated to the acquisition of hind leg-specific morphological traits and the patterning of bristles distribution in honeybees.

A gene regulatory network leading to polyphenism in a social insect was exemplarily investigated by Abouheif and Wray [49] in the ant *Pheidole morrisi* with respect to wing development. In this species, caste determination takes place in three switch points, governed by genetic (the first) and environmental cues (the other two), giving rise to queens, soldiers, and workers, respectively. When analyzing the expression pattern of six genes described as regulators of wing development in *Drosophila*, these authors found that in *P. morrisi* (and in three other ant species) Ubx is always expressed in the hind wing disc/pad during development.

Taken together, these data indicate that the differential expression of Ubx controls alternative appendage development in non-social insects, as well as the acquisition of caste-specific traits in social species, including the honeybee *A. mellifera*.

### Conclusions

This study represents, to our knowledge, the first attempt to understand the molecular mechanisms underlying the caste-specific differential development of the hind leg in honeybees. We propose that this diphenism is driven by a gene regulatory network where crucial switch genes are differentially expressed in workers and queens, such as certain genes encoding cuticular proteins, members of the cytochrome P450 family, as well as a Ubx. In particular, we showed that temporal and spatial differences in Ubx expression during larval and pupal development appear to be a crucial factor for defining divergent hind leg morphogenesis. Furthermore, these findings should provide a conceptual framework to test the function of homeotic genes in honeybee caste development.

## Materials and Methods

### Bees

The honeybee colonies were maintained accordingly to standard beekeeping practices. Workers and queens of the Africanized honeybee *A. mellifera* were collected directly from brood cells. To obtain larvae of the same age, queens were confined for 6 h on combs without young brood. Queen larvae and pupae were obtained by transferring first instar female larvae into queen cups and introducing them back into a regular colony, as described in Barchuk and colaborators [50]. Then, naturally workers were fed with worker jelly and queens with royal jelly, which has substances to induce queen phenotypes such as Royalactin a 57 KDa protein [2]. Worker larvae were staged according to the criteria defined by Michelette and Soares [51], and queen larvae were staged based on Rembold *et al.* [52]. The developmental characteristics of the larvae used in our work are summarized in Table 1.

### Scanning Electron Microscopy

The hind legs from brown-eyed pupae (Pb) of workers and queens were dissected in sterile 0.9% NaCl solution (the pupal cuticle was removed to expose the pharate-adult cuticle) and fixed in Karnovsky’s fixative overnight. Then, the legs were washed in cacodylate buffer and dehydrated using an ethanol washes. After obtaining the critical drying point, the samples were placed on stubs and coated with an ultrathin coating of gold. Samples were visualized and photographed using a Jeol Scanning Microscope JSM-5200 (film ACROS 100/120 Neopan - Fujifilm).

### Organ Sampling and RNA Extraction

Three pooled samples of hind leg imaginal discs of larvae and pre-pupae (n = 20) and hind legs of white-eyed pupae (Pw; n = 6–8), dissected in sterile 0.9% NaCl solution, were used for RNA extraction. Total RNA from pupae was isolated using TRIzol® reagent (Invitrogen) according to the manufacturer’s instructions. As the larvae imaginal discs provided minor amounts of total RNA, it was done using the GenElute™ Mammalian Total RNA Kit (Sigma) according to the manufacturer’s instructions. The total RNA were stored in −80°C until the time of use. RNA quantification was done using Nanodrop-1000 (Thermo Scientific).

### Transcription Profiling Analyses

#### Microarray hybridization

Microarray experiments were performed and are described according to the MIAME specifications [53], and the data have been deposited in the Gene Expression Omnibus database (GEO, at NCBI database) under the accession number GSE34293. The microarrays slides design has been described by [54].

One microgram of total RNA isolated from hind leg imaginal discs of pre-pupae was purified using the RNA Cleanup kit (RNeasy Mini Kit, QIAGEN) and amplified using the Amino Allyl MessageAmp™ II aRNA Amplification Kit (Ambion). Twenty micrograms of amplified RNA was labeled with Cy3 or Cy5 dye (Amersham Biosciences). Two sets of labeled probes were then hybridized to whole genome oligonucleotide arrays (Functional Genomics Unit of the W.M. Keck Center at the University of Illinois, Urbana-Champaign). Prior to pre-hybridization, each slide was UV cross-linked and plunged in 0.2% SDS, water, and ethanol and then centrifuged at a low speed for 3 min. Pre-hybridizations were carried out for at least 45 min in a warm solution (42°C) containing 20% Formamide, 10% Denhardt’s solution 50x, 33.2% SSC 20x, 0.1% SDS and 0.5% tRNA (10 mg/mL) and then rinsed in Milli-Q water, plunged in isopropyl alcohol and dried by centrifugation at low speed for 3 min. Hybridizations were carried out following a loop design with dye-swaps and utilizing two slides. Probes (in 80 µL of 49% Formamide, 49% SSC 20x and 0.2% SDS) were preheated at 55°C for 3 min, placed on microarray slides and covered with lifter-slip cover glasses (22×60, 31.25 µL). Slides were then placed in single slide hybridization chambers and incubated in a water bath for 17 h at 42°C. The washing procedure included the following steps, 2× SSC and 0.1% SDS; 2× SSC; 0.1× SSC and Milli-Q water; shaking manually for 30 s and, all at room temperature. Slides were dried by centrifugation at 2000 rpm for 2 min and scanned using an Axon Genepix 4000B scanner (Molecular Devices) with GENEPIX software 10-micron resolution, Cy3 with Green Laser (532 nm) and Cy5 with Red Laser (635 nm).

#### cDNA synthesis and quantitative RT-PCR (RT-qPCR)

One microgram of total RNA from the hind legs of workers and queens staged at L4, L5F, L5S, L5PP and Pw (prior treated with DNaseI - Invitrogen) was used to synthesize first-stranded cDNA by reverse transcription with SuperScript II Reverse Transcriptase and an oligo (dT_12–18_) primer (Invitrogen).

To quantitatively compare the levels of gene transcription *abd-A*, *atx-2*, *crc*, *dac*, *dll*, *gug*, *RfaBp* and *Ubx* between workers and queens, a Real Time quantitative RT–PCR assay was performed using a 7500 Real-Time PCR System (Applied Biosystems). Amplifications were carried out in a 20-µL reaction mixture containing 10 µL of SYBR® Green Master Mix 2x (Applied Biosystems), 0.8 µL of 10 mM of each gene specific forward and reverse primers (Table 2) and 1 µL of first-strand cDNA samples diluted (1/5) in water. The RT-qPCR conditions were 50°C for 2 min, 95°C for 10 min, followed by 40 cycles of 95°C for 15 s, and 60°C or 62°C (*dac*) for 1 min. Each of the three biological replicates was run in two technical replicates. To choose the reference gene, we tested the *β-actin* (*β-act*) and *ribosomal protein 49* (*rp-49*) genes and the minor expression values were used as references. As described in [55], *rp-49* was the best gene for normalizing gene transcription data and was used in our work. Relative quantities of transcripts were calculated using the comparative Ct method (Applied Biosystems, User bulletin#2). The slope, R^2^ and efficiency values are presented in the Table S2. Statistical analyses were carried out with the SigmaStat 3.1 software (Jandel Corporation, San Rafael, CA, USA), using Two Way ANOVA with two-tailed probabilities, followed by the Holm-Sidak *post hoc* test. The PCR fragment of each gene was cloned, sequenced and aligned using NCBI BLAST tool to check the specificity. All the fragments retrieved the correct and predicted sequence of the gene target.

**Table 2:** Primer sequences, annealing temperatures and fragment lengths (bp).

Primer pairs	Predicted gene	PRIMER sense	PRIMER antisense	Annealing temperature (°C)	Fragment length (bp)
***abd-A***	GB19738	5′ACAACCACTACCTGACGCG	5′ACTCCTTCTTCAATTTCATC	60	114
***atx-2***	GB18802	5′ACAACATCCCAACAGTCAC	5′TGTAGGTCGCAAAGGTAATGG	60	162
***crc***	GB19338	5′GGAGATGTGGAAGCTTGTCA	5′ATGGTTGTACTGGTTGTAAAGT	60	133
***dac***	GB17219	5′GCACCTCAGTCACATGCAAT	5′GACATGTTCGGGTTCACCTT	62	150
***dll***	GB14516	5′ACGCCTACGGATATCACCTG	5′CCCTTTACCGTTCCTCAAG	60	146
***gug***	GB18685	5′ATTAGTTCTGTGACAGAGGAC	5′CATTCCGTACAGAGCAATAAC	60	158
***RfaBP***	GB11059	5′TGCAAAGGCTGACGCTCAC	5′TGCCATCGCTGGTGACAGT	60	167
***Ubx***	GB30077	5′CCCTGGATGGCTATAGCAG	5′GTCAGGCAGAGCGAGTGTG	60	155
***rp-49***	AF441189	5′CGTCATATGTTGCCAACTGGT	5′TTGAGCACGTTCAACAATGG	60	150
***β-actin***	AB023025	5′TGCCAACACTGTCCTTTCTG	5′AGAATTGACCCACCAATCCA	60	156

### Bioinformatics

#### Gene annotation

The genes analyzed in this work were manually annotated, and the primers were designed using the Artemis platform [56]. To confirm primers specificity, the PCR target fragments were sequenced.

#### Microarrays analyzes

Images obtained after scanning hybridized microarrays were processed using ScanAlyze (Rana [http://rana.lbl.gov/EisenSoftware.htm]) with default parameters. All normalizations and fold-change calculations were performed using functions in the library *Limma* of the R/Bioconductor package (R Development Core Team, 2005) as described in Barchuk and others [4]. After statistical analyses (α<0.05; *B*>0) and cutting off values from spots with low intensity signals (compared to empty spots), was selected a list of 200 differentially expressed genes (DEGs). All DEGs with *Drosophila* orthologs were annotated according to the Gene Ontology terms for Biological Process and Molecular Function [57], using the FatiGO web tool [58].

### Immunocytochemistry

Leg staining (DAPI/FP6.87) was performed according to Patel [59], with modifications. The anti-Ubx antibody used herein has previously been used to characterize Ubx expression during honeybee embryogenesis [60] Samples were dissected in sterile 0.9% NaCl and fixed in two different solutions: first with 2% paraformaldehyde in n-heptane and then in 2% paraformaldehyde containing 0.1% Tween-20. Samples were then permeabilized with 0.5 Triton X-100 in PBS and blocked with 0.1% BSA and 5% normal goat serum. Ubx protein expression was detected by incubating legs for 16 hours at 4°C with mouse monoclonal antibody FP6.87 [25], kindly provided by Dr. R. White (University of Cambridge), followed by the addition of Cy3 (Jackson Immunoresearch, 1∶300 dilution) goat anti-mouse. Pupal legs were incubated for 30 hours at 4°C, whereas pre-pupal legs were incubated for 12 hours at 4°C. The negative control was incubated without the primary antibody (see Figure S1). The specimens were washed with 0.5% Triton X-100 in PBS, and DAPI (4′,6-diamidino-2-phenylindole dihydrochloride, Sigma) staining was then performed at room temperature for 4 min, followed by another wash series in PBS with 0.5% Triton X-100. The legs were mounted in 80% Glycerol and analyzed using a Leica TCS-SP5 scanning confocal microscope.

## Supporting Information

Figure S1 **Negative control of immunolocalization of Ubx (FP6.87 antibody) in honeybee worker prepupal hind leg.** **A:** DAPI; **B**: incubated without anti-Ubx antibody (FP6.87); **C**: merge. In blue: DAPI; in red: Ubx; Tar: tarsi; Btar: basitarsi. Original scale bars of confocal system.
(TIF)

Table S1 **List of the top 200 differentially expressed genes between workers and queens’ hind legs at pre-pupal stage.**
(XLSX)

Table S2 **Slope, R2 and efficiency values for each pair of primers used herein.**
(DOCX)
