# Supplementary figures and images for: Hox Gene Expression Leads to Differential Hind Leg Development between Honeybee Castes
Source: PLoS One. 2012 Jul 25;7(7):e40111. doi: 10.1371/journal.pone.0040111 (PMC3405112; doi:10.1371/journal.pone.0040111)

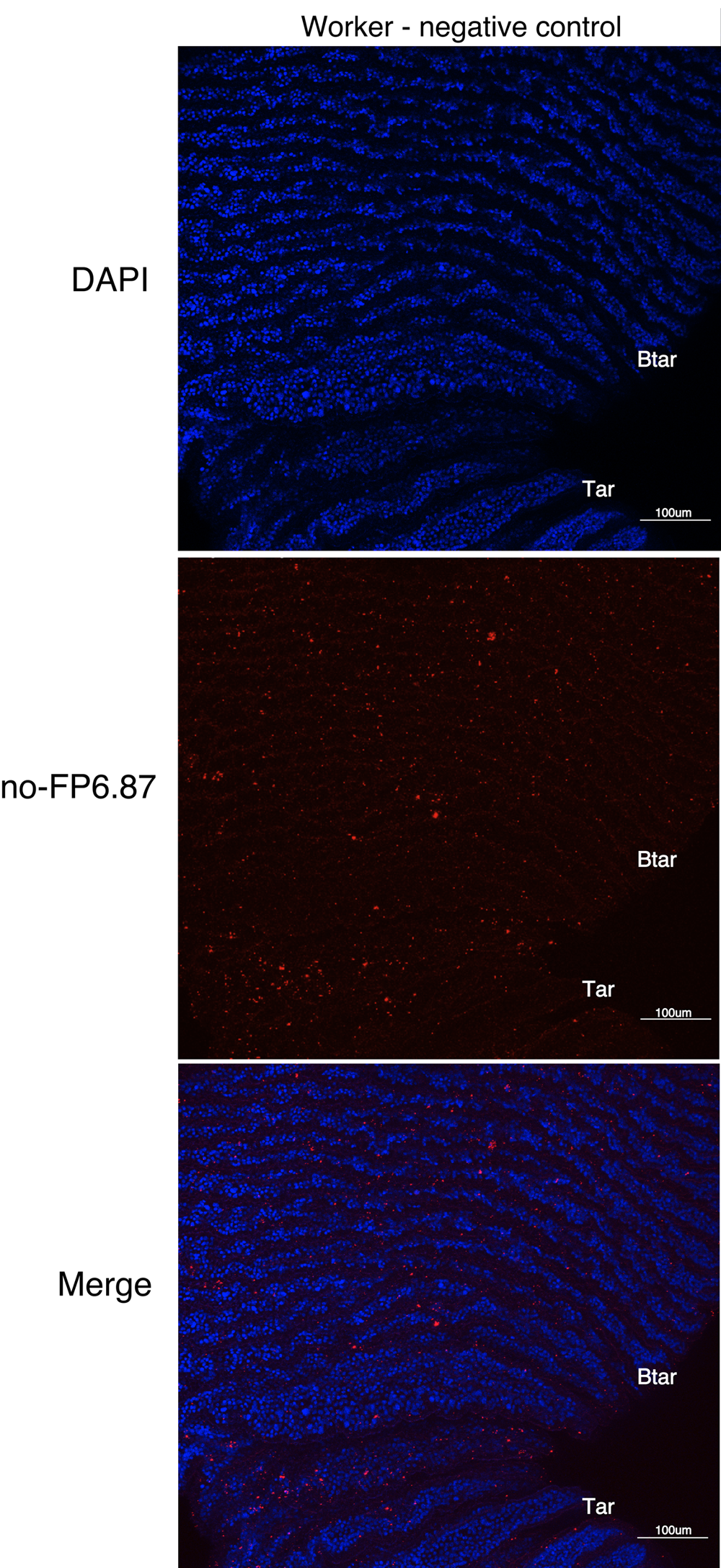

Supplement: Figure S1 — Negative control of immunolocalization of Ubx (FP6.87 antibody) in honeybee worker prepupal hind leg. A: DAPI; B: incubated without anti-Ubx antibody (FP6.87); C: merge. In blue: DAPI; in red: Ubx; Tar: tarsi; Btar: basitarsi. Original scale bars of confocal system. (TIF) [file pone.0040111.s001.tif]
