# Supplementary material for: Hox Gene Expression Leads to Differential Hind Leg Development between Honeybee Castes
Source: PLoS One. 2012 Jul 25;7(7):e40111. doi: 10.1371/journal.pone.0040111 (PMC3405112; doi:10.1371/journal.pone.0040111)
Supplement: Table S2 — Slope, R2 and efficiency values for each pair of primers used herein. (DOCX) [file pone.0040111.s003.docx]

| **Genes** | ***Slope*** | **R2** | **Efficiency (%)** |
| --- | --- | --- | --- |
| ***abd‐A*** | ‐3,007330 | 0,976136 | 100 |
| ***atx‐2*** | ‐3,306692 | 0,996432 | 100 |
| ***crc*** | ‐3,452379 | 0,999311 | 95 |
| ***dac*** | ‐3,266464 | 0,992632 | 100 |
| ***dll*** | ‐3,428257 | 0,998934 | 96 |
| ***gug*** | ‐3,325960 | 0,996484 | 100 |
| ***RfaBP*** | ‐2,977147 | 0,992280 | 116 |
| ***ubx*** | ‐3,236557 | 0,998850 | 100 |
